# Mapping the Possible Futures of Technology and Caregiving

**DOI:** 10.1093/geroni/igaa057.3037

**Published:** 2020-12-16

**Authors:** Joseph Coughlin, Terry Fulmer

## Abstract

As people age, more individuals are expected to need health and caregiver services; it is therefore anticipated that families will face greater demands and carry an increasing burden. Frontier technologies – such as artificial intelligence, sensors and naturalistic user interfaces – are increasingly entering people’s lives, and may be leveraged to support family caregivers. These innovations may make it possible to provide comprehensive care to loved ones while reducing caregiver burden. Similarly, professional caregivers, from social workers to clinicians, may benefit from new devices and services that enable them to provide care more effectively and even proactively prevent a health event. However, challenges around technology maturity, potential risks and skepticism may interfere with a successful implementation. This session reports on research that explores how evolving technologies may shape the future of caregiving. A panel of 41 experts from related disciplines – engineering, data science, medicine, nursing, public policy, business, aging services and more – participated in a modified Delphi study. Across two rounds of data collection, the expert panel provided their opinions and insights about how various technologies and related systems may impact family and professional caregivers by the year 2030. This symposium will present findings from the expert panel, including: how different caregiving tasks and activities may be impacted by new technologies, and what the benefits and barriers may be; possible impacts of new technologies on related topics including elder abuse and fraud, service delivery, and labor and education; and possible future scenarios and expert predictions for 2030.

